# Recommendations to improve use and dissemination of patient versions of oncological clinical practice guidelines in Germany: results of a multi-stakeholder workshop

**DOI:** 10.1186/s12889-024-19893-w

**Published:** 2024-09-03

**Authors:** Nadja Könsgen, Julia Hauprich, Sarah Wahlen, Irma Hellbrecht, Monika Becker, Stefanie Bühn, Nora Meyer, Susanne Blödt, Günther Carl, Markus Follmann, Stefanie Frenz, Thomas Langer, Monika Nothacker, Corinna Schaefer, Dawid Pieper, Jessica Breuing

**Affiliations:** 1 Institute for Research in Operative Medicine (IFOM), Witten/Herdecke University, Cologne, Germany; 2 Institute for Medical Knowledge Management c/o Philipps University Marburg, Association of the Scientific Medical Societies in Germany, Marburg/Berlin, Germany; 3 German Prostate Cancer Support Group, Bonn, Germany; 4 Office of the German Guideline Program in Oncology (GGPO), German Cancer Society, Berlin, Germany; 5 Frauenselbsthilfe Krebs–Bundesverband e.V, Bonn, Germany; 6 German Agency for Quality in Medicine, Berlin, Germany; 7 Faculty of Health Sciences Brandenburg, Brandenburg Medical School (Theodor Fontane), Institute for Health Services and Health System Research, Rüdersdorf, Germany; 8 Centre for Health Services Research, Brandenburg Medical School (Theodor Fontane), Rüdersdorf, Germany

**Keywords:** Patient version of clinical practice guidelines, Oncology, Patient information, Consumer health information, Workshop, World café, Recommendations

## Abstract

**Background:**

Oncological patients have high information needs that are often unmet. Patient versions of oncological clinical practice guidelines (PVG) translate clinical practice guidelines into laypersons’ language and might help to address patients’ information needs. Currently, 30 oncological PVG have been published in Germany and more are being developed. Following a large multi-phase project on oncological PVGs in Germany, recommendations to improve use and dissemination of PVG were adopted in a multi-stakeholder workshop.

**Methods:**

Organisations representing users of PVGs (patients, medical personnel, and multipliers), creators, initiators/funding organisations of PVGs, and organisations with methodological expertise in the development of clinical practice guidelines or in patient health information were invited to participate. The workshop included a World Café for discussion of pre-selected recommendations and structured consensus procedure for of all recommendations. Recommendations with agreement of > 75% were approved, and in case of ≤ 75% agreement, recommendations were rejected.

**Results:**

The workshop took place on 24th April 2023 in Cologne, Germany. Overall, 23 people from 24 organisations participated in the discussion. Of 35 suggested recommendations 28 recommendations reached consensus and were approved. The recommendations referred to the topics dissemination (*N* = 13), design and format (*N* = 7), (digital) links (*N* = 5), digitalisation (*N* = 4), up-to-dateness (*N* = 3), and use of the PVG in collaboration between healthcare providers and patients (*N* = 3).

**Conclusion:**

The practical recommendations consider various perspectives and can help to improve use and dissemination of oncological PVG in Germany. The inclusion of different stakeholders could facilitate the transfer of the results into practice.

## Background

Oncological patients have high information needs that are often unmet [1–4]. Unfulfilled information needs might be related to quality of life, level of depression and anxiety as well as physical symptoms [5, 6]. Patients’ needs range from the basic need for medical information and documentation, to the need for additional information and explanation to complement that provided by health professionals, to the need for support, assistance and advice depending on the difficulties encountered, to the need for listening and psychological support [7]. Patient versions of clinical practice guidelines (PVGs) as a special form of evidence-based practice information might help to address the basic need for medical information and documentation as well as need for additional information and explanation and they often provide contact addresses for additional support. PVGs translate clinical practice guidelines (CPG) into common speech [8]. CPG provide evidence-based recommendations with regard to medical conditions [9] and mainly aim to help health care providers in the decision-making process regarding appropriate care [9–11]. Patients can also use them as a source of information [12, 13]. The transformation into a PVGs helps to increase the understandability for laypersons, since the concept of CPGs is sometimes difficult for them to understand [14, 15]. However, PVGs do not only include a translation of the CPG, they often also contain further and explanatory information. Examples are the introduction to the grading of recommendations, but also background information on the disease comprehensible to patients or further addresses, for example to self-help groups.

In Germany, the German Guideline Program in Oncology (GGPO) develops oncological PVGs for various diseases. Currently, 30 oncological PVG have been published by the GGPO and more are being developed. These PVGs are available in PDF format and as a printed brochure. The printed brochures can be ordered at the website of the German Cancer Aid [16]. The PDF versions can also be downloaded from this website as well as from the website of the GGPO [17]. Both are available free of charge. The development process follows a strict methodology [18].

To our knowledge, there is only scarce information on the use and applicability of oncological PVGs in Germany. To obtain information on this as well as on possible ways for improvements, a large multi-phase study was carried out. The study included a review to assess international methods and approaches of PVGs [19], qualitative interviews on experiences of international guideline producers as well as qualitative interviews and focus groups to analyse the national perspective on the implementation and dissemination of PVGs. Further information on the study can be found in the protocol [20]. The last stage of the study was the development of recommendations based on the previous study results and on the knowledge of several stakeholders within a workshop.

The aim was to formulate recommendations that can improve the use and dissemination of PVGs based on the results of the main project and the consultation of several stakeholders. This will help to transfer the results into practice.

## Methods

### Design

To formulate recommendations for improvement, a one-day workshop was held consisting of a World Café and subsequent voting on the recommendations. The World Café is a method for engaging people in discussions on diverse topics [21]. Unfortunately, we are not aware of any reporting guidelines for publications following a workshop. Accordingly, we did not use any reporting guideline for the preparation of the manuscript.

### Recruitment

Invitations were sent by e-mail to 50 organisations from German-speaking countries. Users of PVGs (patients, medical staff, and multipliers) were included as well as creators and initiators/funding organisations of PVGs and organisations with methodological expertise in the development of clinical practice guidelines or in patient health information. The organisations were selected in cooperation with the project partners: the GGPO, the Association of the Scientific Medical Societies in Germany - Institute for Medical Knowledge Management (AWMF-IMWi), the German Agency for Quality in Medicine (ÄZQ), and two German self-help groups focusing on prostate cancer (Bundesverband Prostatakrebs Selbsthilfe [BPS]) and cancer in women (Frauenselbsthilfe Krebs–Bundesverband [FSH]). A save the date was sent in June 2022, followed by the initial invitation in October 2022, and a reminder in November 2022, if no response was received. If unsuccessful, personal contacts were used where possible. The organisations themselves decided whom to register for participation. However, only one person per organisation could participate.

### Preparation of the workshop

The researchers involved drafted recommendations based on the project results. To achieve this, they summarised the project results. In a brainstorming session, they discussed which recommendations for action can be derived from the results. Some recommendations for action were based on clear results, but in other cases the results contradicted each other. An assessment was made as to which recommendations were based on clear results within the project and whose implementation was considered practicable (feasible) and which were not (to be discussed). The recommendations were then made available to the project partners together with an accompanying explanatory text. The project partners were asked to take part in an online survey to vote on the extent to which the classification in the “feasible” category was appropriate. The recommendations were then re-categorised in line with the voting results, if necessary, and the comments were added anonymously as additional background information. The document, consisting of recommendations, accompanying explanatory text and possible comments from the project partners, was then made available to all workshop participants in advance.

### Workshop

**Fig. 1:**
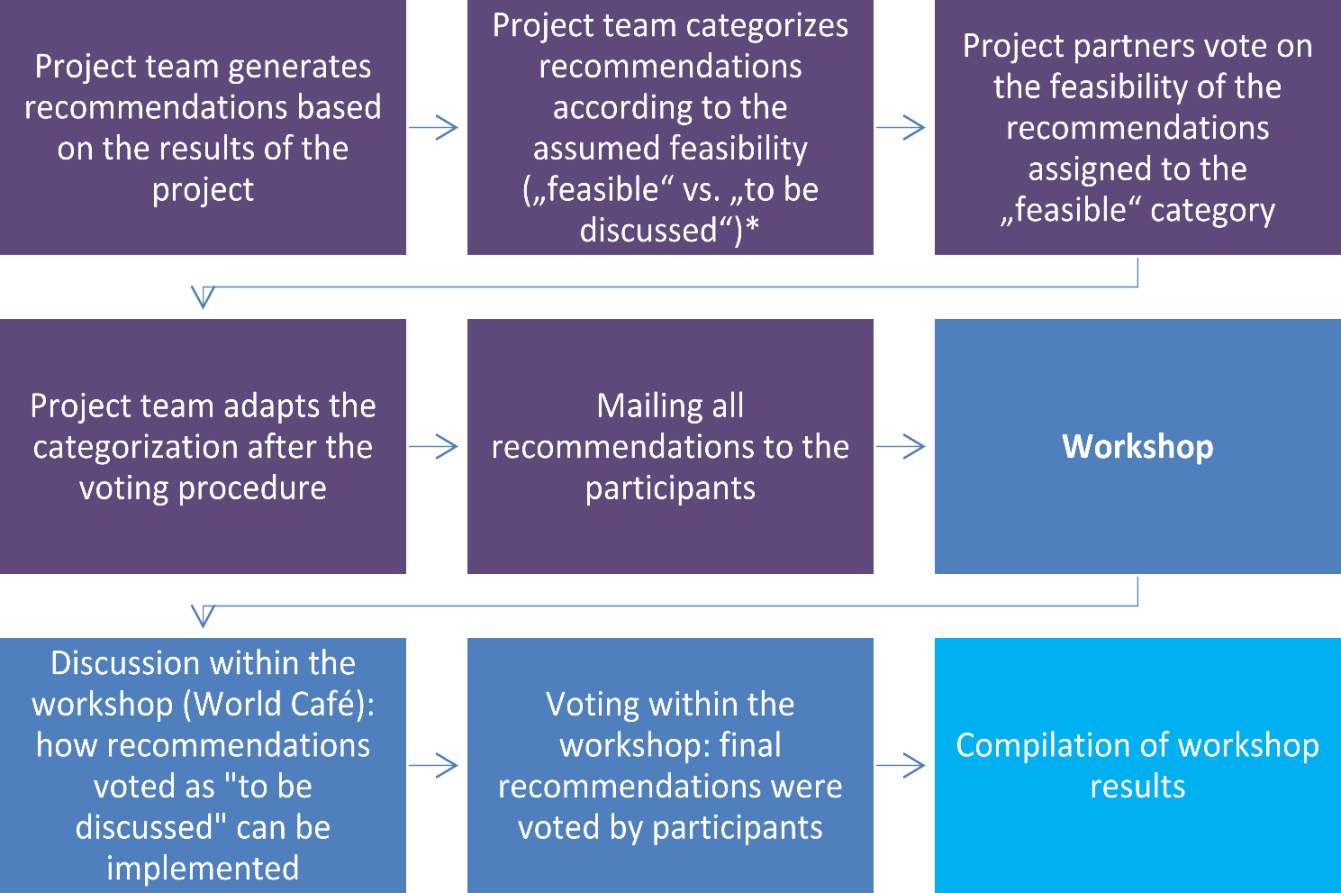
Overall process *category feasible is based on distinct project results and assessed as feasible by the project team; category to be discussed is based on contrary results and/or assessed to be difficult to implement

The workshop was conducted in April 2023. Information on the entire process related to the workshop can be found in Fig. 1.

The Workshop was facilitated by JB (Introduction/Presentation of recommendations and rationale) and MN (neutral moderation/voting). It began with a short introduction of the project and its results to ensure a common knowledge of the topic. After this, the process of the World Café was introduced. Four tables on the topics (1) dissemination, (2) dissemination and use of the PVG in collaboration between healthcare providers and patients, (3) format/design and (digital) links, and (4) digitalisation/up-to-dateness were prepared in advance. Recommendations voted as “to be discussed” in advance were printed as a basis for discussion. Each table was hosted by a member of the project team (JH, NK, SW, JB) to ensure a focused discussion, because of the high number of recommendations assigned to each table. Furthermore, the host wrote down the discussion points on a flip chart and ensured that all group member were involved in the discussion. Participants were assigned to the groups in advance to ensure a heterogeneous composition. After 25 min, the groups switched to the next table. The host summarized the discussion points of the previous groups at the beginning. After the first two tables, there was a lunch break to promote personal exchange. Following the lunch break the discussion was continued until each group had discussed on every topic. After the hosts presented the results of the tables to the whole group, the voting was conducted. Participants voted in blocks on all recommendations from each topic that were a priori assigned to the “feasible” category. Recommendations assigned to the “to be discussed” category were voted individually. Participants could agree, disagree, or abstain from voting. Voting was open using coloured cards. Participants who abstained from voting were excluded from the total population for the calculation of the approval rate. Recommendations with agreement of > 75% were approved, in case of ≤ 75% agreement, recommendations were rejected. If organisations were unable to send a representative or its representative had to leave before voting was conducted for all recommendations, the right to vote could be transferred to the representative of another organisation.

### Data synthesis

Following the workshop, the project team added the discussion points and the voting results to the recommendations developed in advance. The updated recommendations were uploaded to the project website and sent to all workshop participants as well as people who had expressed an interest in the project results.

## Results

### Participants

The five-hour workshop took place on 24th April 2023 in Cologne, Germany. 23 people from 24 organisations participated in the discussion. Four representatives from four organisations had to cancel their participation at short notice for various reasons.

Users of PVGs (patients (*n* = 6), medical staff (*n* = 4), and multipliers (*n* = 2)) were included as well as creators (*n* = 4) and initiators/funding organisations (*n* = 3) of PVGs and organisations with methodological expertise in the development of clinical practice guidelines (*n* = 2) or in patient health information (*n* = 3). In addition, one initiating organisation was not able to send a representative and therefore transferred its voting right for the whole workshop.

### Recommendations on dissemination

In the topic area of dissemination, 13 recommendations were available for voting (three of which were in the “feasible” category). Two out of the 13 recommendations were rejected. The recommendations are shown in Table 1. The use of already existing structures for the dissemination of the PVGs was evaluated very positively. For example, they could be integrated into existing modules of training and continuing education curricula (on communication and evidence-based medicine for service providers (1.4). In addition, to use PVGs could be explicitly mentioned in the requirement catalogue of the oncological centres certified by the German Cancer Society; the current version of the catalogue only refers to patient information in general (1.5). The participants emphasized that this should not displace other high quality information materials. According to the participants, indexing the PVGs for search engine optimisation is very time-consuming because it is a complex technical process (1.6). The use of intuitive terminology on the cover page could already improve the search if necessary (2.4). The participants had a controversial discussion about the use of multilingual information materials such as flyers (1.7). In particular, the use of artificial intelligence was considered beneficial for translation into plain language. When providing information on the PVG in relevant scientific journals, it was assessed important to use free announcements and articles and no advertisement that has to be paid (1.8). Pointing out that congresses aimed at healthcare professionals are already adequately covered using fair stands for information, participants were in favour of presenting PVG at congresses for patients and patient representatives (1.9). The dissemination of the PVG via social media was rejected mainly for the perceived lack of resources (1.11). First, the establishment of structures for the collection of target group-specific media strategies was deemed necessary. The unsolicited sending of flyers and/or printed version of the PVG to relevant healthcare facilities was rejected with the argument that it would be a waste of resources (funds and material; 1.12). The future significance of digital health applications was discussed and partly doubted. Nevertheless, the reference to the PVG in existing digital health applications was evaluated positively (1.13).

**Table 1:** Recommendations on dissemination

No.	Recommendation	Voting
1.1	*Dissemination of the PVG in health care facilities via patient information folders*,* notice boards and visiting services*	*Approved (agreement 100%*, 0 abstentions)*
1.2	*Reference to the PVG in newsletters of the medical societies as well as directly within the corresponding CPG*	*Approved (agreement 100%*, 0 abstentions)*
1.3	*Promotion of the PVG via self-help groups and cancer counselling centers (information events)*	*Approved (agreement 100%*, 0 abstentions)*
1.4	*Integration of the PVG into the training and continuing education curricula for service providers*	*Approved (agreement 100%*,* 1 abstention)*
1.5	*Integration of the PVG into the certification system of the German Cancer Society*	*Approved (agreement 95%*,* 1 abstention)*
1.6	*Indexing the PVGs for search engine optimisation*	*Approved (agreement 79%*,* 6 abstentions)*
1.7	*Promotion of the PVG through posters*,* cards*,* and one-pagers*,* each available in multiple languages*	*Approved (agreement 86%*,* 3 abstentions)*
1.8	*Information on the PVG in relevant scientific journals of the various professional groups*	*Approved (agreement 100%*,* 4 abstentions)*
1.9	*Presence of the PVG at congresses*	*Approved (agreement 100%*,* 0 abstentions)*
1.10	*Prominent positioning of the PVG on the German Cancer Aid website*	*Approved (agreement 100%*,* 3 abstentions)*
1.11	*Dissemination of the PVG via social media*	*Rejected (agreement 0%*,* 6 abstentions)*
1.12	*Unsolicited sending of flyers and/or printed version of the PVG to relevant healthcare facilities*	*Rejected (agreement 75%*,* 4 abstentions)*
1.13	*Reference to the PVG in existing digital health applications*	*Approved (agreement 86%*,* 3 abstentions)*

* “feasible“ category, voted in block

### Recommendations on design and format

In the topic area of design and format, seven recommendations were available for voting (2 of which were in the “feasible” category). Three out of the seven recommendations were rejected. The recommendations are shown in Table 2.

Participants pointed out that product neutrality is sometimes difficult to ensure, especially in the case of photos as distinct from images (2.3). Because the term PVG is not intuitively understandable, intuitive terminology is to be added to the term PVG on the cover page (2.4). Participants controversially discussed proposals for intuitive terminology and pointed out that it should be assessed in advance which terms are understandable for patients. Three recommendations were rejected in view of the high effort that would be involved (2.5–2.7).

**Table 2:** Recommendations on design and format

No.	Recommendation	Voting
2.1	*Clearer presentation of the medical recommendation terms “we recommend”*,* “we suggest”*,* “may be considered” by using bold font*	*Approved (agreement 100%*, 3 abstentions)*
2.2	*Barrier-free presentation of the font*	*Approved (agreement 100%*, 3 abstentions)*
2.3	*Addition of product-neutral images of medical devices*	*Approved (agreement 100%*,* 3 abstentions)*
2.4	*Addition of intuitive terminology to the term PVG on the cover page*	*Approved (agreement 100%*,* 2 abstentions)*
2.5	*Target-group-oriented design of the cover page*	*Rejected (agreement 11%; 5 abstentions)*
2.6	*Option to insert*,* rearrange*,* and remove pages (ring binder)*	*Rejected (agreement 6%; 6 abstentions)*
2.7	*Tab in the margin of the printed version of the PVG + colour differentiation of chapters*	*Rejected (agreement 50%; 12 abstentions)*

* “feasible“ category, voted in block

### Recommendations on (digital) links

In the topic area of (digital) links, five recommendations were available for voting (2 of which were in the “feasible” category). None of the recommendations was rejected. The recommendations are shown in Table 3.

Participants emphasised that recurring cross-references to the explanations of the grading of recommendations are feasible in PDF brochures but not in printed brochures (3.2). They discussed various ways to optimise existing links to target websites (3.4) such as verification on update, annual verification or, in perspective, automated verification. It was assumed that the amount of resources required to create a digital one-pager that lists, among other things, updates to PVG content would be high (3.5). In addition, it was noted that an acceleration of editorial processes is needed to include new content in the one-pager in a timely manner.

**Table 3:** Recommendations on (digital) links

No.	Recommendation	Voting
3.1	*Addition of a QR code on the cover page to link to online PVG formats*	*Approved (agreement 90%*, 2 abstentions)*
3.2	*Recurring cross-references to the explanations of the grading of recommendations*	*Approved (agreement 90%*, 2 abstentions)*
3.3	*Provision of further links (QR codes/active URLs) to additional and more in-depth content*	*Approved (agreement 87%*,* 8 abstentions)*
3.4	*Optimisation of existing links to target websites*	*Approved (agreement 95%*,* 2 abstentions)*
3.5	*Redirection to a digital one-pager that lists*,* among other things*,* updates to PVG content*	*Approved (agreement 80%*,* 3 abstentions)*

* “feasible“ category, voted in block

### Recommendations on digitalisation

In the topic area of digitalisation, four recommendations were available for voting. One of the recommendations was rejected. The recommendations are shown in Table 4.

The participants appreciated the transfer of the content to an app (4.1). There are already plans for implementation. The transfer of the PVG into a digital health application was rejected due to the efforts associated with the benefit assessment in the development of a new digital health application (4.4). In Germany, digital health applications can be prescribed if proven beneficial. With regard to the integration into already existing digital health applications (1.13), the recommendation was rejected as redundant. Linking the PVG to the electronic patient record that is accessible for patients and medical personnel was appreciated (4.3). When linking the PVG to the electronic patient record, some participants emphasised the right of ignorance, so that consent to display the PVG should first be given first. Participants very much welcomed a voice output to make the PVG available to disadvantaged groups of people with physical disabilities (4.2). In case of foreign languages or plain language, a voice output in different languages is needed. According to the participants, this is already feasible for English, but the technology still needs further development for other languages.

**Table 4:** Recommendations on digitalisation

No.	Recommendation	Voting
4.1	*Transfer of the content to an app*	*Approved (agreement 100%*,* 3 abstentions)*
4.2	*Transfer of the (digital) content into a voice output*	*Approved (agreement 100%*,* 9 abstentions)*
4.3	*Linking the PVG to the electronic patient record*	*Approved (agreement 90%*,* 14 abstentions)*
4.4	*Integration/transfer of the PVG into a digital health application*	*Rejected (agreement 5%*,* 6 abstentions)*

### Recommendations on up-to-dateness

In the topic area of up-to-dateness, three recommendations were available for voting. One of the recommendations was rejected. The recommendations are shown in Table 5.

If the underlying CPG is a living CPG, there should be a transition of the PVG to a living PVG (5.1). This requires a simplification of the editorial structures in the development of PVGs. A corresponding simplification is also necessary if there is no living CPG, but the PVG updating process is still to be optimised (5.1.1). Overall, participants again advocated for a general acceleration/optimization of editorial structures in order to integrate new/relevant content into the PVGs more quickly. The display of notifications on the phone must be set individually by the user. Since the organisations that produce PVGs have no influence on this, the recommendation on push notifications (5.2) was rejected.

**Table 5:** Recommendations on up-to-dateness

No.	Recommendation	Voting
5.1	*Transition of the PVG to a living PVG*	*Approved (agreement 95%*,* 3 abstentions)*
*5.1.1*	*Optimisation of the PVG updating process*	*Approved (agreement 95%*,* 3 abstentions)+*
*5.2*	*Push notifications of updated content within app versions*	*Rejected (agreement 67%*,* 9 abstentions)*

+This recommendation was newly formulated during the voting procedure and voted in block together with recommendation 5.1

### Recommendations on use of the PVG in collaboration between healthcare providers and patients

In the topic area of use of the PVG in collaboration between healthcare providers and patients, three recommendations were available for voting (one of which was in the “feasible” category). All recommendations were approved. The recommendations are shown in Table 6.

The distribution of the PVG to patients was considered useful. First, PVG should be offered by physicians (6.2), and the multidisciplinary team should offer it in the subsequent healthcare process (6.3). On the one hand, participants emphasised the improvement of the physician-patient relationship through the implementation of active and/or passive handovers by the physicians as required (6.2); on the other hand, it was stressed that knowledge of the existence of the PVG is a prerequisite for this. Active handover refers to the delivery of the printed PVG, which is briefly introduced in a conversation. Passive handing over refers to the simple handing over of the PVG without any further reference. The latter can be used particularly when physicians feel that an active handover would be overwhelming at this point. However, a passive handover requires an active handover at a later stage. This is separate from the continuous reminder by other health professionals recommended in Recommendation 6.3.

**Table 6:** Recommendations on use of the PVG in collaboration between healthcare providers and patients

No.	Recommendation	Voting
6.1	*PVG first offered by physicians at the time of diagnosis*	*Approved (agreement 100%*, 1 abstention)*
6.2	*Active and/or passive handover of the printed PVG version by physicians as required.*	*Approved (agreement 80%*,* 2 abstentions)*
6.3	*Continuous reoffering of PVG in a multidisciplinary setting*	*Approved (agreement 80%*,* 2 abstentions)*

* “feasible“ category

## Discussion

All in all, 35 recommendations were part of the voting procedure. Of these, 28 recommendations were approved. The recommendations referred to the topics dissemination, design and format, (digital) links, digitalisation, up-to-dateness, and use of the PVG in collaboration between healthcare providers and patients. The recommendations address different stakeholders such as PVG creators, but also healthcare professionals.

Many recommendations refer to the dissemination of PVGs. This is particularly relevant in view of the insufficient awareness observed during the project. A number of participants (patients and healthcare providers) in the qualitative part of study indicated that they appreciated the concept of PVGs but had no awareness about it beforehand [22]. Considering that many participants perceived the PVG as a helpful tool for informed decision-making (data not yet published), the aim should be to increase awareness about the PVGs and their use. To this regard, we provide several recommendations with different approaches addressing patients or healthcare professionals. Different contexts such as training, certification and information policies addressing healthcare professionals are included. Furthermore, providing patient information in healthcare facilities, self-help groups and measures to increase the visibility of PVG in the context of the internet are addressed. A review found that many patients search the Internet for health information and that they most often use a search engine as a starting point [23]. Accordingly, it is important that PVGs can be found well when searching the internet via a search engine. However, many recommendations refer to the integration of PVG into already existing structures. Even though the recommendations provide a practical basis due to the involvement of divergent stakeholders, their implementation in practice is highly important. In this context, further research is needed. For example, a training session to teach physicians on how to integrate PVGs into the doctor-patient conversation could be developed. As a positive side effect, this would also increase doctors’ awareness of the PVGs. Possible obstacles to dealing with the PVGs during the doctor-patient-conversation in detail could be time restrictions experienced by the doctors or the patient-physician relationship. Furthermore, decreased cognitive capacities because of anxiety or stress can also play a role in the ability to perceive information [24]. The timing of the handover might play an important role in this context. According to a qualitative study oncological patients require relevant health information from a very early start [25]. The fact that this time is associated with a high level of emotion, particularly in the case of oncological diseases, can be a challenge in terms of handover. Appropriate training for service providers regarding the PVG could also provide assistance in this regard. After its implementation, such a training session could be integrated in the certification system of the German Cancer Society. This would lead to a higher awareness and use of the PVG on the physicians’ as well as the patients’ side.

The implementation of some recommendations would also enable people to use PVG who were previously unable or only partially able to do so due to various circumstances. In our recommendations, we refer to non-native speakers as well as people with impaired vision. There are further groups of people such as patients with intellectual disabilities [26] who may not be addressed by the PVGs. Because our project did not provide any results on this, they are not mentioned in the recommendations. Nevertheless, we want to emphasize the importance of addressing all target groups. This is certainly very challenging due to highly divergent needs. Information needs differ in terms of what information is needed, in what form and in what level of detail. A survey found that the Internet is the most frequently sought source of health information by both men and women [27]. However, the frequency of searching on the Internet also depends on underlying sociodemographic factors such as the socioeconomic status. In the course of a systematic review, it was found that information needs (of patients, relatives and the general population) vary in type and scope [28]. Beyond topics such as treatment, diagnosis, prevention and health promotion, aetiology, and prognosis, where information needs are high, information on topics such as rehabilitation and impact on social life was in demand less frequently. In this context, it would be helpful to individualise PVGs to a greater extent. The user test of a PVG also showed that some needs are so heterogeneous that individualisation, if possible, should be attempted [29]. If all potential information needs were addressed in a PVG, the scope would be far too large for many patients. This trade-off is a challenge that might be addressed by (digital) links or the use of different formats. The integration of PGVs in different formats such as apps or the electronic patient record could enable a staggered integration of the content.

At least at present, the implementation of a part of the recommendations is only possible for some of the formats offered. One example is the recurrent explanation of the grading of recommendations. This is easy to implement for PVGs in PDF format, but difficult in case of a printed PVG. However, printed PVGs continue to be very popular even though there might be differences between patient populations (e.g. age [22]).

Especially in the context of digitalisation, there are likely to be some opportunities for further development of PVGs in the future. Some of them are directly taken into account in our recommendations; others have been discussed in the context of the implementation of individual recommendations. This was the case, for example, with the recommendation to optimize existing links to target internet sites. Participants mentioned that this could be done automatically in the future.

### Strength and limitations

Some limitations have to be considered when interpreting our results. Even though the list of participants is not exhaustive, the big players in the German field of PVGs and patient information took part. Due to time constraints, the recommendations from the “feasible” category could not be discussed in detail during the World Café. In the course of the voting procedure, there was restricted additional time for discussion, if necessary. Nevertheless, it became apparent that there was no need for discussion for most of the recommendations from the feasible category. However, for some of the other recommendations more discussion time would have been helpful. On the other hand, we assume that the time restriction could increase the participation rate by allowing the participants to arrive and depart on the day itself. The World Café was chosen to enable all stakeholder to participate in the discussion and to share their point of view. On the other hand, this left less time for discussion in the plenary session.

Additionally, some participants of the workshop gave the feedback, that they would have preferred an anonymous online voting procedure. Since there were also some delays in counting, especially when people changed their minds or needed longer time to think about it, the project team would use online voting procedure in the future.

In the course of the discussion, some participants expressed that they did not want to prescribe to specific addressees (e.g. German Cancer Aid) what they must do. The moderator then clarified that these were only recommendations and not obligations. Nevertheless, it cannot be ruled out that this misinterpretation may have influenced the voting behaviour of some participants.

Our project referred to oncological PVG only, therefore the majority of recommendations can only be applied to oncological PVG. This becomes clear, for example, in the recommendation 1.5 (integration of the PVG into the certification system of the German Cancer Society) or 1.10 (Prominent positioning of the PVG on the German Cancer Aid website) since they are specifically targeted at relevant stakeholders in the field of oncology. However, a number of the recommendations without named addressees also clearly belong to the field of oncological PVG. One example is recommendation 2.1 including the clearer presentation of the medical recommendation by using bold front. For oncological PVG, unlike other PVG, an italicized font has been used to date. However, this was described by some participants as not striking enough. Other recommendations may also apply to non-oncological PVG. Since the concept of PVGs was not well known, it can be assumed that this is a fundamental circumstance and not exclusively related to the field of oncology. Accordingly, it should be investigated to what extent measures to disseminate PVG, for example, can be implemented beyond oncology.

Considering all this, the project achieved practical recommendations under consideration of various perspectives. This can help to improve use and dissemination of (oncological) PVGs in Germany.

## Conclusion

Overall, 35 recommendations were part of the voting procedure. Of these, 28 recommendations were approved. The recommendations referred to the topics dissemination, design and format, (digital) links, digitalisation, up-to-dateness, and use of the PVG in collaboration between healthcare providers and patients. The practical recommendations consider various perspectives and can help to improve use and dissemination of (oncological) PVGs in Germany.

## Data Availability

Most of the data generated or analysed during this study are included in this published article. Further details are available from the corresponding author on reasonable request.

## Abbreviations

CPG: Clinical Practice Guidelines
GGPO: Office of the German Guideline Program in Oncology c/o German Cancer Society
PVG: Patient version of clinical guideline
